# Galectins in Glioma: Current Roles in Cancer Progression and Future Directions for Improving Treatment

**DOI:** 10.3390/cancers13215533

**Published:** 2021-11-04

**Authors:** Samy Ajarrag, Yves St-Pierre

**Affiliations:** INRS-Centre Armand-Frappier Santé Biotechnologie, Laval, QC H7V 1B7, Canada; samy.ajarrag@inrs.ca

**Keywords:** galectin, brain tumors, blood–brain barrier, immunotherapy, glioblastoma

## Abstract

**Simple Summary:**

Glioblastomas are among the most common and aggressive brain tumors. The high rate of recurrence and mortality associated with this cancer underscores the need for the development of new therapeutical targets. Galectins are among the new targets that have attracted the attention of many scientists working in the field of cancer. They form a group of small proteins found in many tissues where they accomplish various physiological roles, including regulation of immune response and resistance to cell death. In many types of cancer, however, production of abnormally high levels of galectins by cancer cells can be detrimental to patients. Elevated levels of galectins can, for example, suppress the ability of the host’s immune system to kill cancer cells. They can also provide cancer cells with resistance to drugs-induced cell death. Here, we review the recent progress that has contributed to a better understanding of the mechanisms of actions of galectins in glioblastoma. We also discuss recent development of anti-galectin drugs and the challenges associated with their use in clinical settings, with particular attention to their role in reducing the efficacy of immunotherapy, a promising treatment that exploits the capacity of the immune system to recognize and kill cancer cells.

**Abstract:**

Traditional wisdom suggests that galectins play pivotal roles at different steps in cancer progression. Galectins are particularly well known for their ability to increase the invasiveness of cancer cells and their resistance to drug-induced cell death. They also contribute to the development of local and systemic immunosuppression, allowing cancer cells to escape the host’s immunological defense. This is particularly true in glioma, the most common primary intracranial tumor. Abnormally high production of extracellular galectins in glioma contributes to the establishment of a strong immunosuppressive environment that favors immune escape and tumor progression. Considering the recent development and success of immunotherapy in halting cancer progression, it is logical to foresee that galectin-specific drugs may help to improve the success rate of immunotherapy for glioma. This provides a new perspective to target galectins, whose intracellular roles in cancer progression have already been investigated thoroughly. In this review, we discuss the mechanisms of action of galectins at different steps of glioma progression and the potential of galectin-specific drugs for the treatment of glioma.

## 1. Introduction

Glioma is the most common primary intracranial tumor, representing more than 80% of malignant brain tumors [1]. In 2007, the World Health Organization classified the main glial tumor into four grades, from the least to the most aggressive tumor subtype. Grade 1 tumors include pilocytic astrocytomas, pleomorphic xanthoastrocytomas, and subependymal giant cell astrocytomas; grade 2 tumors include more common infiltrating gliomas, such as grade 2 oligodendrogliomas and astrocytomas; grade 3 includes tumors such as anaplastic oligodendrogliomas, anaplastic astrocytomas, anaplastic oligoastrocytomas, and anaplastic ependymomas; and grade 4 tumors, which include glioblastomas (GBMs) [2,3]. GBM represents approximately half of all gliomas [1].

The current standard of care for patients with glioma is not curative but comprises multimodal therapy that includes elective surgery, radiotherapy, and treatment with temozolomide (TMZ), an oral DNA alkylating agent known to induce cell cycle arrest at G2/M. Unfortunately, the response rate remains relatively low. For patients with low-grade glioma, the average survival time is approximately 5–8 years, with death occurring following progression to high-grade glioma. Successful development of an effective therapy faces a number of obstacles that include the relative inefficiency of drugs in terms of their crossing the BBB, intratumoral heterogeneity, and intrinsic resistance to drug-induced cell death. Novel therapeutic alternatives include treatment with bevacizumab, a monoclonal antibody directed against vascular endothelial growth factor (VEGF). This antibody is increasingly being used with some success, although the benefit of this drug remains somewhat controversial [4]. Additional strategies based on the use of immune checkpoint inhibitors (ICIs) are also being explored. However, again, they have not yet resulted in a significant survival benefit [5]. Indeed, there is increasing evidence that glioma treatment faces another obstacle: a strong local immunosuppressive tumor microenvironment (TME). Accordingly, many efforts have recently been made to understand the immunosuppressive factors involved, at either the cellular or molecular level. These efforts are complementary to those that are focused on increasing the bioavailability of drugs after they cross the BBB.

In this review, we discuss the emergence of galectins as potential targets for the treatment of GBM. While research on galectin started in the 1970s, only in the last 10–15 years have we begun to grasp its critical role in cancer development, particularly in brain cancer. This new knowledge, together with the concomitant development of new generations of galectin inhibitors and drug delivery technologies, offers new opportunities for the development of new and effective treatments for patients with glioma.

## 2. Galectins and Their Implication in Cancer Progression

The discovery of galectins goes back more than 40 years, following the discovery of a β-D-galactoside-binding protein from tissue homogenates of electric eel by Vivian Teichberg [6]. In the decades that followed, many more galectins were identified in many species, from invertebrates to humans [7,8]. Today, galectins are known as a structurally related family of animal lectins that show a high level of evolutionary conservation and that can accomplish multiple intracellular and extracellular functions during development as well as various physiological processes, including regulation of the immune response. All galectins harbor a carbohydrate recognition domain (CRD) consisting of approximately 130 amino acids, within which we found a glycan-binding site (GBS) that allows them to bind β-galactosides and other monosaccharide scaffolds. Based on the number of CRDs and their quaternary structure, galectins have been divided into three distinct groups: (1) prototypic galectins, which possess a single CRD that can form homodimers; (2) the tandem repeat type, consisting of a single polypeptide encoding two CRD domains joined by a peptide linker; and (3) a chimera-type galectin, which has a single C-terminal CRD linked to a non-CRD N-terminal peptide chain rich in proline and glycine. It is important to note that while all galectins possess a CRD domain, this does not endow them with a de facto ability to bind sugars. The classical example is galectin-10 (Gal-10), which is known to form Chardot–Leyden crystals in many tissues, particularly eosinophils [9]. Gal-10 has recently been shown to bind eosinophil granule cationic ribonucleases in a carbohydrate-independent manner [10] but is best known for its critical role in asthma, as Gal-10 crystals induce chronic inflammation of airways [11]. We now know that Gal-10 is not the only galectin that does not bind β-galactosides. Another example is galectin-13 (Gal-13), which has a unique structure formed by a homodimer stabilized by disulfide bonds and is unable to bind lactose [9]. In fact, over the years, many studies have shown that galectins are also endowed with the ability to bind intracellular and extracellular ligands via carbohydrate-independent interactions [12,13,14,15,16]. As we discuss later, these findings have major implications for the development of galectin-specific drugs, which have mostly been targeting the GBS and the carbohydrate-dependent functions of galectins. However, before addressing the challenges associated with the development of galectin-specific drugs, let us review their implications in the progression of GBM, focusing on the most promising targetable galectins. For readers unfamiliar with the general and multiple roles of galectins in other types of cancer, we invite them to read the recent review by Girotti and colleagues [17].

## 3. The Case of Galectin-1

Gal-1 was the first galectin identified and is still one of the most studied galectins, with more than 2000 published reports to date, a third of them over the last 5 years. The first reports that Gal-1 might be a valuable target for the treatment of glioma were published 20 years ago, with many studies by the group of Robert Kiss and colleagues in Brussels. This group published landmark studies in the field of glioma showing that the expression of Gal-1 in glioma correlated with the aggressiveness of the tumor and low survival [18,19,20]. They showed that Gal-1 was preferentially expressed in the tumor margin, consistent with a possible role in migration and GBM invasiveness. Expression of Gal-1, however, is not restricted to GBM. It has been observed in several types of brain cancer [19,21]. Very recent studies have confirmed that abnormally high levels of Gal-1 correlate with poor survival, most notably in patients with GBM undergoing adjuvant radiotherapy [22]. Interestingly, this abnormally high level of Gal-1 can be detected in the serum of patients, thereby providing an interesting tool for identifying patients at risk and who could benefit from Gal-1-directed therapies.

The above reports on high expression levels of Gal-1 in patients with GBM provided a rationale for elucidating its role in GBM. Early mechanistic studies focused on the role of Gal-1 in cell migration. Knockdown of *LGALS1* in glioma cells indicated that Gal-1 controls multiple genes related to motility and migration, including genes encoding CapG and Map-2, two regulators of actin cytoskeleton dynamics, cadherin 6, as well as other proteins, such as Adam15, is known to interact with α9β1 integrin to modulate intercellular adhesion and cellular invasiveness [23,24,25,26]. More recently, Gal-1 has been shown to interact with FAM289, an oncogenic protein expressed in glioma, and to promote its translocation from the cytosol to the nucleus [27]. The role of Gal-1 in promoting the invasiveness of glioma cells is not restricted to its intracellular functions, as other reports have shown that Gal-1 can promote cell invasion by promoting the adhesion of cells to proteins of the extracellular matrix, such as fibronectin and laminin, and to cell surface β1 integrins [28,29,30,31]. These findings were consistent with observations showing that Gal-1-dependent cell migration occurs through activation of the RhoA/ROCK pathway, which causes a rearrangement of the cell actin skeleton to promote migration and invasion [32,33]. Another series of studies has shown that Gal-1 may not only be involved in the control of cellular invasiveness of GBM cells but may also be responsible for conferring resistance to chemotherapy and radiotherapy. A review published in 2010 by Le Mercier and colleagues discussed in detail this mechanism of action (MoA), which implicates intracellular Gal-1, whose expression is triggered via hypoxic conditions [34]. To our knowledge, however, this association between Gal-1 and chemoresistance in GBM has not been studied thoroughly since, although several studies published in other cancer models are consistent with this possibility [35,36,37,38,39,40].

**Figure 1:**
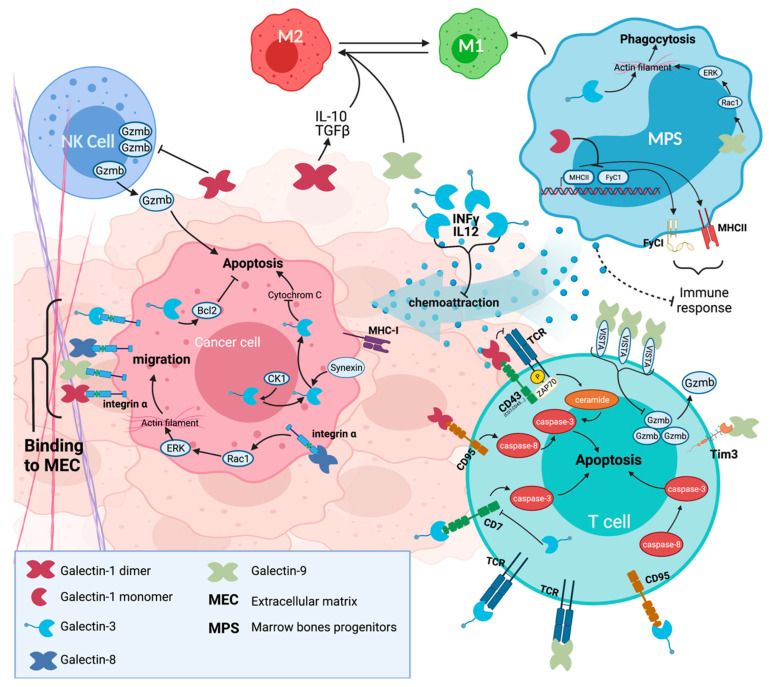
Multiple roles of intra- and extracellular galectins in glioma.

Considering the recent development and success of immunotherapy to stop cancer progression and the well-documented role of Gal-1 in the control of the immune response, most notably against cancer killing immune cells, it is reasonable to predict an increased interest in investigating whether inhibition of Gal-1 may help to improve the success rate of immunotherapy against glioma. This possibility has been well documented recently [41,42,43,44]. Studies of various types of cancer, including glioma, have revealed that the immunosuppressive effect of Gal-1 could be mediated by several mechanisms of action (MoA), such as promoting infiltration of immunosuppressive Tregs [45], polarization of macrophages towards an ant-inflammatory phenotype [46], inhibition of the capacity of NK cells, through the miRNA-TLR7-IFNbeta pathway, to kill cancer cells [47,48], or deletion of activated cancer killing T cells following binding of extracellular Gal-1 to glycoreceptors expressed on various subpopulations of immune cells (Figure 1) [49,50,51]. In the latter case, this binding induces apoptosis of activated immune cells, as observed by the landmark paper by Linda Baum’s group, who first reported that Gal-1 induces activated T cell death [49]. Gal-1 might also play a role in T cell regulation via the induction of Gal-9. In head and neck cancer, it has been observed that Gal-1 promotes cellular expression of PD-L1 and Gal-9 at the cell membrane, contributing to early tumor evasion of the T cell-mediated immune response [52]. Moreover, Gal-1 knockdown has been shown to enhance the efficacy of ICI and dendritic cell-based immunotherapy [52,53,54]. Taken together, there is accumulating support for the idea that targeting Gal-1 represents a valuable strategy for overcoming local immunosuppression associated with the TME of GBM.

## 4. The Case of Galectin-3

Galectin-3 (Gal-3) was first described in 1983 under the name CBP35 [55]. Since its discovery, chimera-type Gal-3 has been the subject of more than 5000 published reports, making it the most studied member of the galectin family. The chimera type Gal-3 has a short 12-amino-acid N-terminus that contains a serine phosphorylation site that is responsible for its subcellular translocation, a 110-amino-acid-long collagen-alpha-like stretch that is rich in proline, alanine, and glycine, and a 140-amino-acid C-terminal domain encoding the CRD. Gal-3 has been shown to be a versatile protein involved in several biological processes, including cell adhesion, cell activation and chemoattraction, cell growth and differentiation, the cell cycle, mRNA splicing apoptosis, and a multitude of other cellular functions [56,57,58,59]. Such diverse cellular functions are explained, at least in part, by its distinct cellular localization [60].

**Figure 2:**
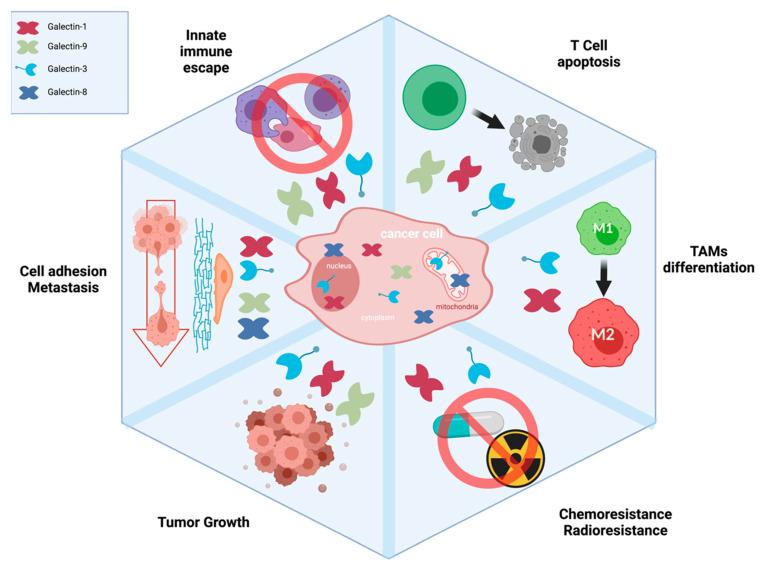
Impact of galectins on the hallmarks of cancer.

The possible implication of Gal-3 in the progression of glioma has been studied for decades, albeit at a very slow pace, but has received increased attention over the last 10 years. Gal-3 is expressed in astrocytic cells and microglia, although most of the attention has been paid to mesenchymal subtype of GB’ [61,62,63,64]. A common finding with Gal-3 in GBM is that overexpression is associated with a poor prognosis and correlates with malignant potential [64,65,66]. As in the case of Gal-1, its abnormally high level of expression seems to be induced by hypoxia and other stress signals [64,65,66,67,68]. From a mechanistic point of view, the studies on Gal-3 have contributed to the paradigm that galectins, aside from their role in the regulation of the immune response, have multiple roles in cancer progression (Figure 2). Studies on its MoA were initially focused on its role in the invasiveness of glioma cells and chemoresistance [34]. This role in invasiveness has recently been confirmed in an elegant 3D in vitro model, which revealed a potentially critical Gal-3\β1 integrin interaction for promoting single-cell migration and, by extension, a possible role in metastasis [69]. New indications that Gal-3 may mediate chemoresistance in GBM have also been reported in recent years. Using both in vitro and in vivo models, Ikemori and colleagues [70] showed, for example, that Gal-3 knockdown in human U87MG cells delayed tumor engraftment and reduced tumor growth, providing support for the possibility of targeting Gal-3 to sensitize glioma cells to cell death. Whether such inhibition of Gal-3 perturbs intracellular pathways that were initially suspected of mediating resistance to cell death, such as the Bcl-2 [71], Ras [72], or CD95 pathways [73], or requires phosphorylation of Gal-3 on critical residues [74] remains unclear.

Needless to say, given the immunosuppressive activity of galectins in general, we cannot ignore the potential implication of Gal-3 in contributing to the immunosuppressive TME found in GBM [57,75,76]. This implication can be summarized in at least three distinct immune-related functions: recognition of tumor antigens, regulation of apoptosis, and neutralization of cytokines. For example, secreted Gal-3 directly modulates the recognition of antigens by the T cell receptor (TcR) expressed by CD8-positive effector T cells via its ability to form extracellular lattices, thereby distancing the TcR from the costimulatory CD8 receptor [77]. Inhibition of Gal-3 by N-acetyllactosamine, a natural ligand of galectins, restores TcR–CD8 colocalization. Whether this role is shared by other galectins remains unclear. Another MoA involved in Gal-3-mediated immunosuppression and shared by other galectins is its ability to induce apoptosis of activated immune cells. The role of Gal-3 in inducing apoptosis has been discussed in more than 500 publications. Another recently described MoA by which Gal-3 regulates the local immune response is via its interaction with chitinase-3-like-1 protein, a glycoprotein secreted by a variety of cancer cells, increasing its ability to reprogram monocyte-derived macrophages into a protumor-M2-like immunosuppressive phenotype [78]. Finally, another MoA that has not received a lot of attention is the ability of Gal-3 to trap and neutralize key cytokines that are essential for the development of an effective immune response. This was reported for the first time by the group of Pierre Van der Bruggen. His group showed that accumulation of secreted Gal-3 in the TME leads to the capture of glycosylated IFNγ and IL-12 [79]. Whether this role is restricted to Gal-3 or shared by other galectins is unclear. However, overall, all of these immune-related functions have provided the rationale for testing whether Gal-3 can potentiate the effect of ICIs. A recent study using belapectin, a polysaccharide polymer that contains galactose and other sugars, has reported that this drug can potentiate the effect of ICIs and anti-OX-40 agonists in preclinical studies of multiple cancer models [80,81,82]. Notably, a recent in silico analysis by Takashima and colleagues showed that the genes encoding Gal-3 and Gal-9 were among the top 20 immunotherapy-related genes whose expression was significantly modulated. Surprisingly, however, another study has shown that lower overexpression of both *LGALS3* and *LGALS9* was associated with a poor prognosis [68]. Although these results could be of importance in establishing a gene signature for prognostic purposes, they underline the complexity of Gal-3, whose functions are dependent on its intra- and extracellular localization as well as its posttranslational localization (e.g., phosphorylation).

## 5. The Case of Galectin-8

Galectin-8 (Gal-8) is a tandem-repeat type of galectin that was discovered by Hadari and colleagues in 1995 [83,84]. Gal-8 controls many functions associated with cell adhesion, migration, proliferation, and cell survival. In lung cancer, Gal-8 is able to modulate cell adhesion and migration by binding integrin and signaling toward the FAK pathway [85]. Some suggest that Gal-8 promotes cell migration via an interaction with ALCAM at the cell surface [86]. This hypothesis has received support in a breast cancer model that showed that concomitant silencing of Gal-8 and ALCAM delays tumor growth [86]. Others suggest that Gal-8 might induce cell spreading and migration by binding integrins and promoting sustained activation of the ERK pathway, leading to actin filament reorganization [87]. Accordingly, further study showed that in T cells, Gal-8 binds to integrins and induces Rac1, leading to actin rearrangement through the ERK pathway. This promotion of cell spreading seems to be shared by a large spectrum of cell types, including cancer cells, and could modulate a wide range of T cell-driven immune processes [88]. Nevertheless, Gal-8 is now best known for its role in autophagy since the publication of a landmark paper by Teresa Thurston in 2012 showing that it acts as a danger signal and protects cells from damage caused by intracellular bacteria by modulating the integrity of endosomal and lysosomal compartments [89]. Subsequent studies showed that such a protective mechanism concerns not only bacteria but also viruses [90]. For example, Gal-8 has been shown to act as a matrix protein capable of tuning cell adhesion by ligating and inducing clustering of several surface receptors, such as integrins, in different cell types [85,91,92]. To our knowledge, however, whether Gal-8 has the ability to modulate tumor growth via an autophagy-dependent mechanism has not been studied. Autophagy plays a central role in cancer progression [93]. In glioma, autophagy is believed to be an adaptive MoA used by glioma cells to protect them from unfavorable conditions [94].

Thus, could Gal-8 be involved in glioma progression? Could it be a potential target? The answers to these questions are not entirely clear, although there are some indications that warrant more investigations. To our knowledge, the only report to address these questions directly was published by Metz and colleagues. Using recombinant human Gal-8 and shRNA-mediated silencing experiments in an in vitro model system of glioma (U87), the authors showed that Gal-8 increased cell migration, proliferation, and resistance to cell death [95]. Whether these observations translate into other glioma models and, most importantly, in vivo has not been addressed in the literature since.

One interesting avenue that has not been investigated in glioma is the role of Gal-8 in inducing the secretion of cytokines by endothelial and cancer cells, such as SDF-1 and MCP-1 [96]. This has been shown in many tumor models. The ability to increase the secretion of SDF-1 (CXCL12) is particularly interesting. A recent review on the role of CXCL12 describes in detail how this chemokine accumulates in the TME and leads to resistance to ICIs, while inhibitors of this cytokine increase the effectiveness of ICIs [97]. In an in vivo model of breast cancer, Correia and colleagues recently showed that this accumulation of CXCL12 in the TME is necessary for inducing NK cell-mediated tumor dormancy [98]. This MoA may explain why inhibition of CXCL12 and its receptors (CXCR4) increases the response to ICIs [99,100].

Despite these interesting studies on the role of Gal-8, it is important to mention that its role in cancer progression is not black-and-white. Although similar to other galectins, Gal-8 can induce apoptosis in immature T cells [101,102], recent studies in various experimental systems by Maria Tribulatti’s group suggest that Gal-8 has important immunostimulatory functions in peripheral naïve T cells and promotes the uptake of antigens by antigen-presenting cells [103,104]. Interestingly, Gal-3 has been shown to inhibit the immunostimulatory effect of Gal-8 on naïve T cells [105], emphasizing the need to develop galectin inhibitors that are very specific.

## 6. The Case of Galectin-9

Gal-9 was first described by Türeci and all in 1997 [106] as a tandem repeat type of galectin with two CRDs linked by a peptide of approximately 40 kDa, but its size varies according to the isoform [107,108]. Gal-9 is expressed in mesenchymal subtype GBM subtype and more likely to be found in the core of the tumor, unlike its counterpart Gal-1 that is expressed mainly in margin cells [20,109]. While Gal-9 has been studied for many years, it has received increasing attention since it was recognized as a ligand for T cell immunoglobulin and mucin domain-containing protein 3 (TIM-3) [110]. The authors showed that Gal-9 binds to TIM-3 via its GBS and induces the death of Th1 (but not Th2) cells by both apoptotic and necrotic mechanisms and that injection of recombinant Gal-9 in mice does indeed reduce the number of IFNγ-secreting Th1 cells. Interestingly, subsequent studies have shown that interfering with the TIM-3 and PD1/PDL1 pathways is more effective than targeting each pathway alone in restoring antitumor immunity [111,112]. Since then, the Gal-9/TIM-3 axis has received special attention from scientists working in the field of cancer immunotherapy and is now considered a mechanism of resistance when using ICIs in cancer patients [113,114,115,116]. The functional relationship between Gal-9, TIM-3, and ICIs was confirmed by a recent study showing that Gal-9 also binds to PD1 and plays a major role in the resistance of TIM-3-positive T cells to cell death [117]. The authors of this study further showed that a therapy that combines anti-Gal-9 and anti-PDL-1 blocking antibodies resulted in better survival than monotherapy. Targeting Gal-9 is thus certainly an avenue to explore in the case of glioma. We indeed know now that Gal-9 is expressed in glioma and that its expression varies with tumor progression and cell type and is associated with TIM-3 [109,118,119]. A recent report showed that patients with glioma with high expression of Gal-9 have a higher chance of developing malignant tumors and that Gal-9 is an independent indicator of poor prognosis [120]. Moreover, GBM tumors have subpopulations of cells that coexpress Gal-9 and PD-L1, suggesting a close relationship between Gal-9 and the PD-1 pathway in glioma [118]. Gal-9 expression also correlates with increased M2-associated macrophages, consistent with prior reports showing that Gal-9 is able to induce the M1-to-M2 transition to promote tumor growth [109,121,122]. Overall, the exciting development that followed the initial identification of Gal-9 as a ligand for TIM-3 introduces a promising new era for cancer research on glioma.

## 7. Approaches for Targeting and Delivery of Galectins

Since the recognition of galectins as critical factors that regulate tumor progression, particularly the immunosuppressive TME, researchers have focused on developing galectin inhibitors for several decades. Although preclinical studies have provided some hope for the use of these inhibitors in human clinical trials, efforts have generally not produced the kind of success one would hope for. Development of highly selective galectin-specific drugs that minimize off-target effects has indeed been impaired by the striking structural similarities between the GBSs of different galectins. In most cases, these drugs are high-molecular-weight inhibitors, synthetic or naturally occurring polysaccharides that are used to specifically block the binding of extracellular galectins (mostly Gal-1 and Gal-3) to carbohydrate structures on cell surface receptors [123,124]. Achieving specificity and high affinity for these compounds is, however, a formidable task. As in the case of Gal-1, the development of inhibitors against Gal-3 has been focused mostly on the use of saccharide-based drugs, such as GCS-100, GR-MD-02, and TD139 [125,126,127]. However, some of these inhibitors have been shown to potentiate the effect of ICIs. This is the case for GR-MD-02 (also called belapectin), which has shown promising results in a Phase I study with metastatic melanoma and head and neck squamous cell carcinoma [80]. Another inhibitor of Gal-3, the monosaccharide GB1107, has also been shown to increase the efficacy of anti-PD-L1 to inhibit tumor growth in a mouse xenograft model with the lung adenocarcinoma cell line (A549) [82]. *GB1107* is an inhibitor of Gal-3, with a K_d_ of 37 nM for human galectin-3, suggesting that inhibition of galectins does not require an affinity in the picomolar range. Whether these effects were solely mediated by inhibition of Gal-3, however, is not yet clear. GR-MD-02, for example, has similar affinities for human Gal-1 and Gal-3 (K_d_ of 8 and 10 μM, respectively) [128]. GB1107, on the other hand, is relatively specific for Gal-3 [129]. Some of these inhibitors, such as GR-MD-02 (~50 kDa) have a relatively high molecular weight. It is noteworthy to mention that although the affinity of carbohydrate-based inhibitors is generally in the micromolar range, which is often considered low for an inhibitor, they have proven to be very effective in inhibiting the function of galectins in vitro and in vivo. A case in point is the in vivo effect of belapectin [80,81,82]. Another example is the recently described monosaccharide galactose-based antagonist of human Gal-8. This inhibitor has an affinity in the low micromolar range and is capable of inhibiting the secretion of cytokines by breast cancer cells [130].

To add to the challenge of developing specific galectin drugs for the treatment of glioma is the need to cross the BBB. However, recent advances in this area suggest that this is not insurmountable. Recent seminal discoveries on the presence of afferent and efferent routes (between the brain and regional lymph nodes, such as the deep cervical lymph nodes) have shown that the CNS immune privilege paradigm might be overstated [131]. Many consider that in terms of the immune response, the brain should be considered a distinct rather than privileged site [132]. Support for this view is provided by increasing evidence that activated T cells in the periphery can gain access to the brain and that the BBB is not hermetically sealed, as we previously believed [131]. Nevertheless, the number of infiltrating immune cells within brain tumors is relatively low, possibly because brain tumors are considered “cold” due to the presence of a strong immunosuppressive TME. There is indeed a relatively clear consensus on the fact that the GBM microenvironment is a highly immunosuppressive milieu of tumor cells and immune cells. Although the cause of this immunosuppression is likely to be multifactorial [133], it is becoming clear that galectins play an important role in regulating the immune response. It is likely that inhibition of one or more galectins may induce a cascade-like mechanism that could, at least partially, transform cold tumors toward a more inflammatory phenotype [133].

Notwithstanding the hermeticity of the BBB, one logical option is to use siRNA-based therapeutics [134]. The potential of galectin-specific siRNA in increasing the survival of mice with glioma has been shown by Danhier and colleagues using a combination of EGFR and Gal-1-specific siRNA combined with TMZ using chitosan lipid nanocapsules that were injected intracranially [135]. The authors showed that this treatment significantly reduced the expression of both proteins and prolonged the survival of mice implanted with human U87 glioma cells. It is important to note that the athymic nude mice used in the study had no defect in the non-MHC-restricted response and that the cytotoxic activity of NK cells was usually significantly higher in the athymic nude mice than in the normal mice. This issue was addressed in another study by Van Woensel and colleagues [136] using an immunocompetent mouse model and GL261 murine GBM cells. The authors not only confirmed the benefit of targeting Gal-1 but also confirmed the role of Gal-1 in controlling the immunosuppressive TME. Interestingly, the authors exploited a nose-to-brain delivery strategy to bypass the hermeticity of the BBB. They further showed that Gal-1-specific siRNA synergized with ICIs to augment the survival of tumor-bearing mice. Taken together, these results provide strong support for the possibility of targeting galectins for the treatment of glioma. A very interesting study has recently shown that the benefit of targeting Gal-1 in GBM may not be solely associated with its immunosuppressive role. Shanarek and colleagues have indeed shown that inhibition of Gal-1, by OTX008 in this case, interferes with the intracellular role of Gal-1 [137]. The authors have shown that OTX008, a calixarene derivative, prevents the interaction between Gal-1 and HOXA5 and the ensuing on chemoresistance and proliferation of GBM. These results supported previous data showing that OTX008 inhibits proliferation of GBM lines, including U87 and cancer cell lines derived from patients with GBM [138]. Such an intranuclear function of Gal-1 is not overly surprising, as galectins are well known to accomplish various roles in different subcellular compartments, including the nucleus [139].

In addition to siRNA-based therapeutics and chemical drugs, a logical option for targeting galectins would be to use antibodies, which have proven their efficacy in immunotherapy in patients with different types of cancer, including GBM [140]. It is well known, however, that less than 0.1% of conventional antibodies in serum can penetrate the brain through the BBB. An interesting alternative would be to use smaller (12–15 kDa) single-domain antibodies, such as variable domain of heavy-chain antibodies (VHHs), also called Nanobodies^®^ (Nbs), a name registered by Ablynx, a company working specifically on these proteins. A unique feature of Nbs is their extended convex-shaped paratope, which can recognize epitopes that are normally inaccessible to conventional Abs. This is possible because their hypervariable region is made of a single stretch of a.a. residues composed of flexible peptide loops, including a relatively long complementary determining region (CDR)-3 loop that is extended and comprises 15–25 residues on average (compared with 12 residues in humans). The structure of their antigen-binding region is thus ideally suited for targeting epitopes buried at the dimer interface of prototypic galectins, such as the dimer interface, or even epitopes located at the deep end of the GBS. Another critical consideration is that Nbs usually have very high affinity for their ligands, and similar to Abs, they are usually highly specific. The humanized form of Nbs can also be generated by genetic engineering, thereby limiting their immunogenicity. Interestingly, in contrast with conventional Abs, Nbs can be produced using a prokaryotic expression system. This is a non-negligible advantage considering the high cost of immunotherapy using conventional Abs. Because of their small size, not surprisingly, a number of teams have recently studied whether Nbs can cross the BBB and whether they could be used for the treatment of brain diseases. The results are very encouraging and have shown that Nbs can reach the brain via the BBB either directly, via receptor-mediated transcytosis, or indirectly, using, for example, various forms of carriers, including nanoparticles or liposomes [141,142]. We also better understand why some Nbs are more efficient at crossing the BBB. For instance, Nbs with a basic isoelectric point can form the blood–brain barrier in vivo after peripheral injection, without the need for any invasive procedure that weakens the BBB [143]. Ongoing studies have shown that such VHH can be used for either inhibition of specific cancer targets, for delivery of active payloads, or for imaging purposes in GBMs [143,144,145,146]. The development of Nbs as therapeutics in general, however, is only in its infancy. The first Nbs for the treatment of a human disease (in this case, acquired thrombotic thrombocytopenic purpura) was approved by the FDA and European agencies in 2018. To our knowledge, there are currently no clinical trials aimed at testing the efficacy of Nbs for the treatment of GBM.

## 8. Conclusions

In summary, there are accumulating and strong indications that galectins are among the emerging therapeutic targets for the treatment of brain tumors. The success of galectin-specific treatments will depend, however, on our ability to develop novel and specific therapeutic drugs that are compatible with Trojan-horse-like delivery strategies capable of overcoming the challenges associated with the presence of the BBB and the immunosuppressive TME.

## Abbreviations

BBB	blood–brain barrier
GBM	glioblastoma
MoA	mechanism of action
TMZ	telozolomide
ICI’s	immune checkpoint inhibitors
VEGF	vascular endothelial growth factor
GBS	glycan-binding site
CRD	carbohydrate recognition domain
TME	tumor microenvironment
Nbs	nanobodies
